# Association of carcinoid tumor and low grade glioma

**DOI:** 10.1186/1477-7819-10-236

**Published:** 2012-11-08

**Authors:** Emeline Tabouret, Maryline Barrié, Cecile Vicier, Anthony Gonçalves, Olivier Chinot, Patrice Viens, Anne Madroszyk

**Affiliations:** 1 Département d’Oncologie Médicale, Institut Paoli-Calmettes, 232 Boulevard Ste Marguerite, 13009, Marseille, France; 2 Service de Neuro-Oncologie, Assistance Publique des Hôpitaux de Marseille, Hôpital de le Timone, Marseille, France

## Abstract

**Background:**

Lung carcinoid tumor and low grade glioma are two uncommon malignancies.

**Patients and methods:**

We report the case of 24-year-old man who presented with respiratory disease. Imaging investigations showed a right lung tumor and histological analysis confirmed a typical carcinoid tumor. As part of initial staging, brain MRI revealed an asymptomatic right frontal lesion. First, a right pulmonary lobectomy was performed without adjuvant treatment. In second time, brain tumorectomy was performed. Histological examination confirmed the diagnosis of low grade glioma (LGG). The patient remained in complete remission 2.5 years after the initial diagnosis.

**Results:**

This is the first case reporting the association between LGG and lung carcinoid tumor, while no association between LGG and a systemic tumor have been published to date. Association of lung carcinoid tumor with other malignant diseases has been reported but remained uncommon. Only minimal data support a potential molecular common origin.

**Conclusion:**

This exceptional association may be fortuitous. However, their concomitant diagnoses suggest a potential association between both rare diseases. A genetic susceptibility remains possible.

## Background

Carcinoid bronchopulmonary tumors represent approximately 1 to 2% of all lung neoplasms. Low grade glioma (LGG) are rare, diffuse, slow-growing, primary neuroectodermal tumors that occur in the central nervous system. In this report, we present the first association between LGG and lung carcinoid tumor. We also performed a comprehensive review of the literature on the association between LGG or carcinoid tumor, and systemic malignancies.

## Case presentation

**Figure 1:**
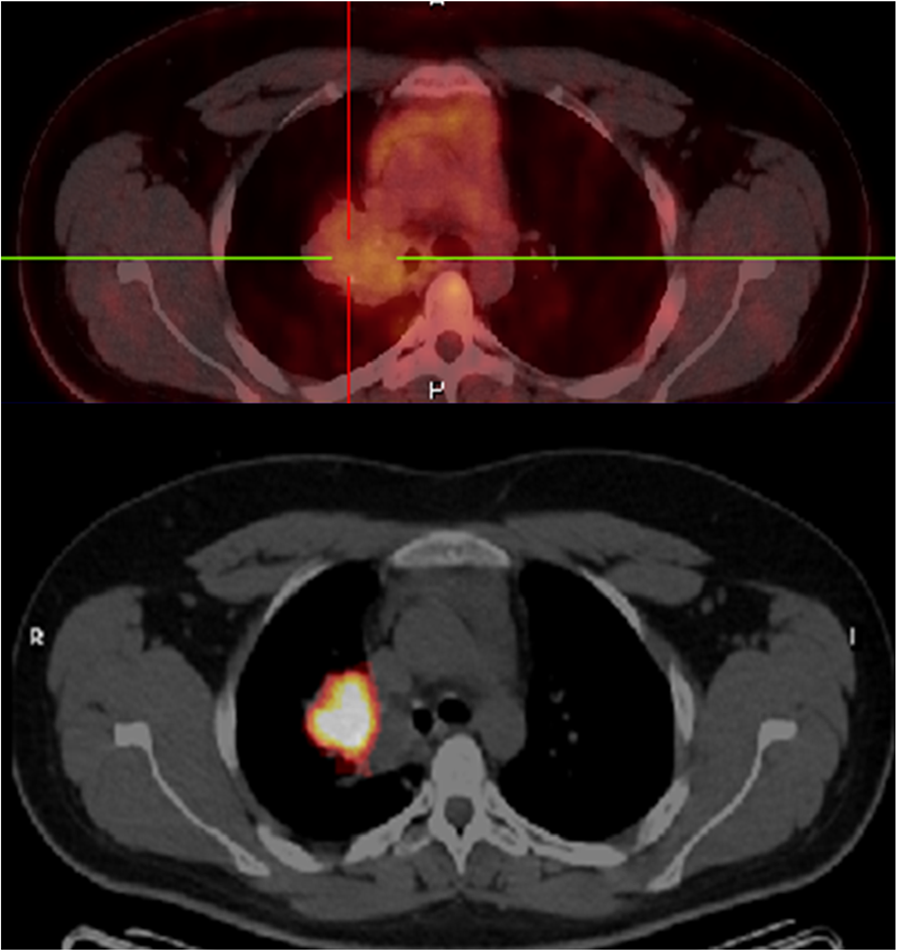
Lung TEP scanner (top) and octreoscanner (bottom).

**Figure 2:**
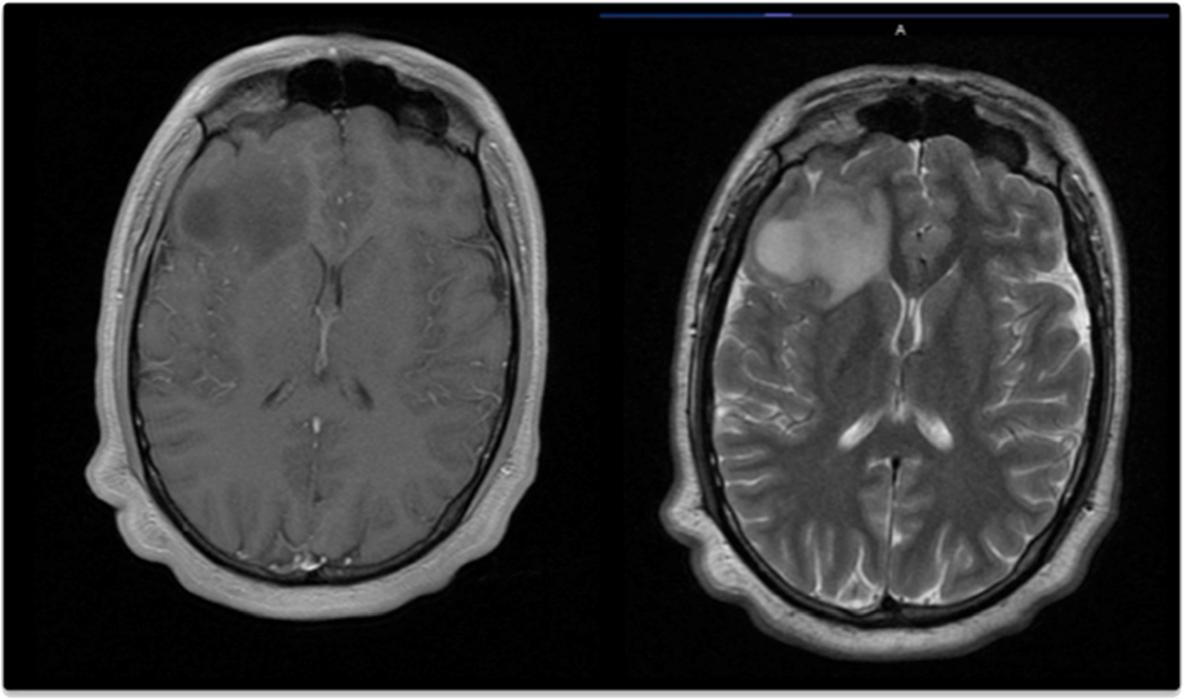
Brain magnetic resonance imaging (MRI): T1 gadolinium sequence (left) and T2 sequence (right).

A 24-year-old man presented in September 2009 with respiratory symptoms including cough and hemoptoic expectorations, without altered general status. Past medical history was restricted to Oto-Rhino-Laryngological viral infections without smoking, alcohol, or asbestos exposure. Clinical observation was not relevant with normal respiratory and neurological examinations. Thoracic radiography showed a mass in the right lung. Computed tomography (CT) confirmed a lung tumor (56 mm) in the right superior lobe associated with mediastinal lymph node involvement without visceral metastasis. First, lung fibroscopy was performed. It was complicated by massive hemoptysis. Histological analysis concluded a diagnosis of typical carcinoid tumor. Positron emission tomography was moderate positive (Standardized Uptake Value = 4) and octreoscan presented an intensive fixation for lesions previously described on the CT scan (Figure 1). Both examinations were negative for brain lesions. The plasmatic rate of insulinemia, gastrinemia, chromogranine A, cyfra21 and Neuron-Specific Enolase (NSE) were normal. The rate of plasmatic serotonin was 365 μg/L (normal: 100 to 300 μg/L). The plasmatic \ hCG and β-hCG levels were normal. To complete disease evaluation, brain magnetic resonance imaging (MRI) was performed and revealed a right frontal infiltrative lesion that exhibited hyposignal T1 and hypersignal in T2 and flair sequences, with no contrast enhancement on T1 gadolinium, compatible with an LGG (Figure 2).

Lung surgery consisted of a right superior lobectomy with a resected tumor that measured 5 cm. Lymph node involvement was restricted to one of the 16 explored nodes. Definitive histology confirmed a typical carcinoid tumor pT3N1M0 R0 (Stage IIIA). After multidisciplinary consultation no adjuvant treatment was indicated.

On subsequent surgery, gross total resection of the brain tumor was performed. Anatomopathologic examination concluded a diagnosis of grade II oligoastrocytoma. Further immunohistochemical and molecular analyses identified expression of p53, lack of MGMT (O(6)-methylguanine-DNA methyltransferase) promoter methylation, absence of IDH (Isocitrate Dehydrogenase) mutation and no amplification of EGFR (Epidermal Growth Factor Receptor). Using Fluorescence In Situ Hybridization technique, no 1p19q codeletion was detected. An adjuvant treatment with radiotherapy (60Gy) and concomitant temozolomide (75mg/m^2^ per day, five days per week, during 6 weeks) was performed. At present, patient is routinely follow-up and complete remission is maintained 2.5 years after the initial diagnosis.

Oncogenetic examination was proposed to the patient. It showed an atypical aggregation of oncologic diseases but did not find genetic abnormality or hereditary risk factor. No additional biological investigation was performed, as this tumor association did not refer to a known genetic syndrome. Only blood conservation was realised.

## Discussion

To our knowledge, this is the only reported case of an association between a carcinoid tumor and glioma. This exceptional association may be fortuitous. However their concomitant diagnoses may suggest a potential relationship between these two both rare tumors.

Carcinoid tumours are a unique class of malignancies capable of producing hormones identical to those from the nervous system [1]. Its representatives are found in the lung, gastrointestinal tract, skin, thyroid, thymus, pancreas, biliary and urogenital tracts. Lung carcinoid tumors tend to occur at a younger age as in our patient [2]. Survival is generally good, especially in patients with early stage disease, with reported 5-year survival rates of 44 to 97% [3,4].

Low grade gliomas are tumors that exhibit glial differentiation without aggressive findings (angiogenesis). Oligoastrocytomas exhibit a mixed phenotype in which some tumor cells show astrocytic differentiations while others show oligodendroglial differentiations [5]. No association between low grade glioma and systemic tumor were reported.

Association of carcinoid tumors with other malignant diseases is uncommon. Rare isolated cases have been reported with association between carcinoid tumors and other carcinomas (gastric [6], renal [7], colorectal, and breast carcinoma [8]) [9]. Two cases of synchronous pulmonary carcinoid tumor and non-Hodgkin’s lymphoma have been described [10]. Carcinoid tumors of the lung were also rarely described in the multiple endocrine neoplasia type 1 (MEN1) [11]. MEN1 is an autosomal dominant genetic disorder first described in 1903 by Jacob Erdheim, which has been associated with a combination of more than 20 endocrine and nonendocrine lesions. The prevalence of carcinoid bronchial tumors among MEN1 is only 2%. Tumors associated with MEN1 include meningiomas and ependymomas but not glioma [11]. So, no evidence of clinical and genetic association between primary brain and carcinoid tumors was found.

Two molecular analyses showed correlation between LGG and carcinoid tumors. The first reported that astrocytomas could share N-CAM-related antigens with small lung carcinomas. This biological observation could support a relationship or potential common origin between neural and neuroendocrine tumors [12]. A second publication compared gene expressions of small cell lung cancers, pulmonary carcinoid tumors and oligodendrogliomsa using high-density cDNA arrays [13]. They found similarity of gene expression among carcinoid tumors and oligodendrogliomas, suggesting that pulmonary carcinoids are related to neural crest-derived brain tumors. These molecular similarities and presumed common tumoral origin of both carcinoid and LGG raise the possibility of a unique molecular abnormality driving the development of the two tumors in our patient. However, explorations of genetic causes in this patient remain unsuccessful to date.

## Conclusion

Ours is the first case reporting the association between low grade glioma and lung carcinoid tumor. The occurrence of these tumors might be an as yet undescribed association that is more than fortuitous. A genetic susceptibility remains possible.

## Consent

Written informed consent was obtained from the patient for publication of this report and any accompanying images.

## Competing interests

The authors declare that they have no competing interests.

## Authors’ contributions

ET acquired and analysed data and drafted the manuscript. MB, CV, AG, OC, PV partyicipated to the acquisition of data. OC helped to draft the manuscript. AM supervised this case report, participated in the acquisition of data and helped to draft the manuscript. All authors read and approved the final manuscript.
